# Interaction of Potent Mitochondrial Uncouplers with Thiol-Containing Antioxidants

**DOI:** 10.3390/antiox8060194

**Published:** 2019-06-23

**Authors:** Ljudmila S. Khailova, Alexander M. Firsov, Elena A. Kotova, Yuri N. Antonenko

**Affiliations:** Belozersky Institute of Physico-Chemical Biology, Lomonosov Moscow State University, 119991 Moscow, Russia; khailova@genebee.msu.ru (L.S.K.); amfamf@yandex.ru (A.M.F.)

**Keywords:** mitochondrial uncoupler, FCCP, fluazinam, membrane potential, respiration rate, isolated mitochondria, reactive oxygen species, thiol-containing antioxidants, planar bilayer lipid membrane

## Abstract

It is generally considered that reactive oxygen species (ROS) are involved in the development of numerous pathologies. The level of ROS can be altered via the uncoupling of oxidative phosphorylation by using protonophores causing mitochondrial membrane depolarization. Here, we report that the uncoupling activity of potent protonophores, such as carbonyl cyanide 4-(trifluoromethoxy)phenylhydrazone (FCCP), carbonyl cyanide 3-chlorophenylhydrazone (CCCP), and fluazinam, can be abrogated by the addition of thiol-containing antioxidants to isolated mitochondria. In particular, N-acetylcysteine, glutathione, cysteine, and dithiothreitol removed both a decrease in the mitochondrial membrane potential and an increase in the respiration rate that is caused by FCCP. The thiols also reduced the electrical current that is induced by FCCP and CCCP across planar bilayer lipid membranes. Thus, when speculating on the mechanistic roles of ROS level modulation by mitochondrial uncoupling based on the antioxidant reversing certain FCCP and CCCP effects on cellular processes, one should take into account the ability of these protonophoric uncouplers to directly interact with the thiol-containing antioxidants.

## 1. Introduction

At present, there is a common opinion that the oxidative stress is causative of a plethora of serious diseases, including neurodegenerative disorders, heart failure, kidney pathologies, and endocrine system malfunction. According to a series of papers [1,2,3,4,5,6,7,8], the uncoupling of oxidative phosphorylation in mitochondria can significantly affect the generation of reactive oxygen species (ROS). In view of these results, the modulation of the ROS level by uncouplers was considered as the basis of their protective action against numerous diseases [9,10,11]. Researchers used to present data on the effect of antioxidants on the uncoupler-induced protection to prove the ROS-associated mechanism of the therapeutic activity of uncouplers. In particular, based on the complete removal of the cardioprotective effect of carbonyl cyanide 4-(trifluoromethoxy)phenylhydrazone (FCCP) by N-acetylcysteine (NAC) [12], the authors attributed the cardioprotection to the influence of FCCP on the level of ROS. Furthermore, the involvement of ROS level modulation by carbonyl cyanide 3-chlorophenylhydrazone (CCCP), FCCP, and fluazinam in certain cellular processes was validated by the sensitivity of the uncoupler impacts on these processes to NAC [13,14,15,16,17]. The authors [18] even suggested that ROS regulate mitochondrial transmembrane potential based on the data that NAC inhibited depolarization of mitochondria in cells by CCCP. 

On the other hand, a series of studies revealed evidence that CCCP and FCCP can both covalently react with thiols [19,20,21]. Of note, the blocking of CCCP action by aminothiols was reported in very early works [22,23,24].

Among the long list of mitochondrial uncouplers, the fungicide fluazinam is known not only for its extremely high uncoupling potency, but also for a peculiar property, namely, fast disappearance of the activity after the addition of the uncoupler to isolated mitochondria [25,26,27]. The rapid deactivation of fluazinam was found to be associated with an effect of endogenous glutathione on a 3-chloro substituent in the phenyl moiety, obviously with nucleophilic substitution of the chlorine atom by a glutathione residue [25]. However, no data regarding the effect of exogenously added antioxidants on the fluazinam depolarizing activity in mitochondria have been reported so far.

Here, we performed a thorough investigation of functional consequences of the interaction of CCCP, FCCP, and fluazinam with thiol antioxidants, in particular, their impact on the ability of these uncouplers to decrease mitochondrial membrane potential and accelerate respiration of isolated mitochondria. We also demonstrated the suppression of the protonophoric activity of CCCP and FCCP in model lipid bilayer membranes.

## 2. Materials and Methods

### 2.1. Materials 

Most chemicals, including CCCP, FCCP, fluazinam (3-Chloro-N-(3-chloro-5-trifluoromethyl-2-pyridyl)-α,α,α-trifluoro-2,6-dinitro-p-toluidine), 2,4-dinitrophenol (DNP), tyrphostin A9 (3,5-Di-tert-butyl-4-hydroxybenzylidenemalononitrile), niclosamide, tetrachlorosalicylanilide, NAC, glutathione (GSH), dithiothreitol (DTT), rotenone, diphytanoylphosphatidylcholine (DPhPC), and safranine O were from Sigma. Tetrachlorotrifluoromethylbenzimidazole (TTFB) was a gift of Lev Yaguzhinsky (Moscow State University).

### 2.2. Isolation of Rat Liver Mitochondria 

Mitochondria were isolated from rat liver by using differential centrifugation [28], according to a slightly modified procedure previously described [29]. The animals were handled and experiments were performed in accordance with the international guidelines for animal care and use and the Institutional Ethics Committee of A.N. Belozersky Institute of Physico-Chemical Biology at the Lomonosov Moscow State University approved them (protocol #3 on 12 February 2018).

### 2.3. Mitochondrial Respiration 

The respiration of isolated rat liver mitochondria was measured at the mitochondrial protein concentration of 0.8 mg/mL by using a Clark-type oxygen electrode (Strathkelvin Instruments, UK), as described previously [29].

### 2.4. Membrane Potential (ΔΨ) Measurement in Isolated Mitochondria

The mitochondrial membrane potential (ΔΨ) was evaluated from the difference in the absorbance at 555 and 523 nm (ΔA) of the safranine O dye [30] measured with an Aminco DW-2000 spectrophotometer, as described previously [29]. Mitochondria were incubated in the medium containing 250 mM sucrose, 5 mM MOPS, 0.5 mM KH_2_PO_4_, 1 mM EGTA, 2 μM rotenone, 5 mM succinate (pH 7.4), 1 μg/mL oligomycin, and 15 μM safranine O at the mitochondrial protein content of 0.6–0.9 mg protein/mL.

### 2.5. Planar Bilayers 

Bilayer lipid membranes (BLMs) were formed by the brush technique [31] from a 2% solution of diphytanoylphosphatidylcholine in n-decane. The measurements of electrical current through BLM were performed, as described previously [29].

## 3. Results and Discussion

**Figure 1:**
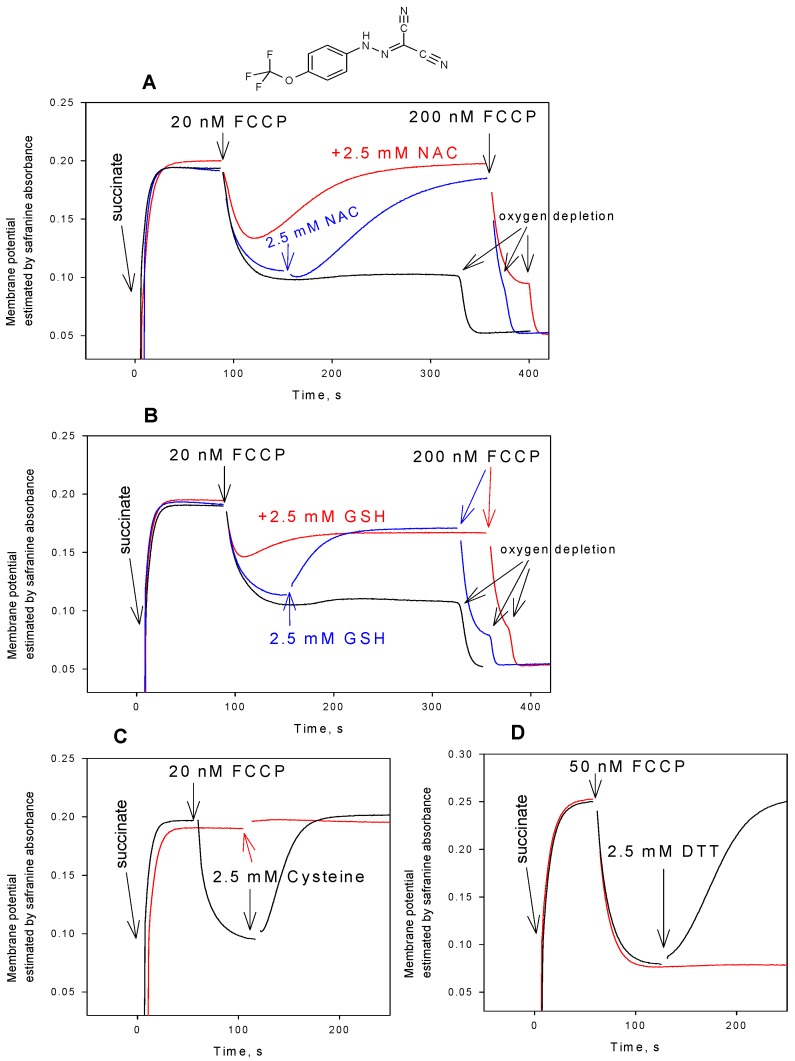
(**A**) Effect of N-acetylcysteine (NAC, 2.5 mM) on the uncoupling activity of carbonyl cyanide 4-(trifluoromethoxy)phenylhydrazone (FCCP) in rat liver mitochondria. (**B**) Effect of glutathione (GSH, 2.5 mM) on the uncoupling activity of FCCP in mitochondria. (**C**) Effect of cysteine (2.5 mM) on the uncoupling activity of FCCP in mitochondria. (**D**) Effect of dithiothreitol (DTT, 2.5 mM) on the uncoupling activity of FCCP in mitochondria. The uncoupling activity was estimated by the mitochondrial membrane potential measurements with safranine O (15 µM). Y-axis shows absorbance of safranine at 555 nm minus absorbance at 523 nm. Red curves corresponded to the addition of N-acetylcysteine (NAC) (2.5 mM, panel A) or GSH (2.5 mM, panel B) prior to mitochondria. Chemical structure of FCCP is shown on the top of the Figure. For other conditions, see Materials and methods.

**Figure 2:**
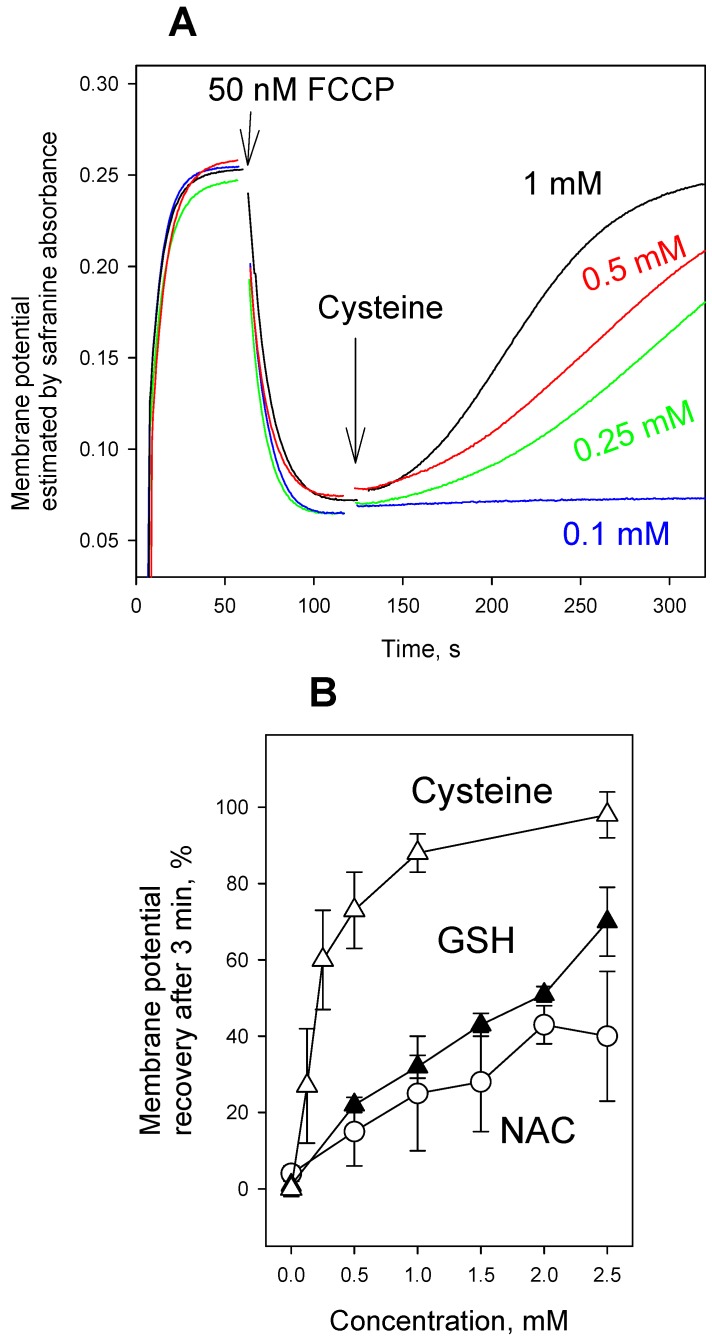
(**A**) Effect of various concentrations of cysteine on the uncoupling activity of FCCP (50 nM) in rat liver mitochondria estimated by the mitochondrial membrane potential measurements with safranine O (15 µM). Y-axis shows absorbance of safranine at 555 nm minus absorbance at 523 nm. (**B**) Dose dependence (shows Mean ± S.D., n = 3) of the recovery of the membrane potential 3 min. after the addition of NAC, GSH, or cysteine. For other conditions, see Materials and methods. Of note, when added at millimolar concentrations, cysteine and dithiothreitol caused the complete abrogation of the FCCP-induced decrease in mitochondrial membrane potential, while the effects of GSH and NAC were much slower and sometimes incomplete even if measured longer than 3 min.

**Figure 3:**
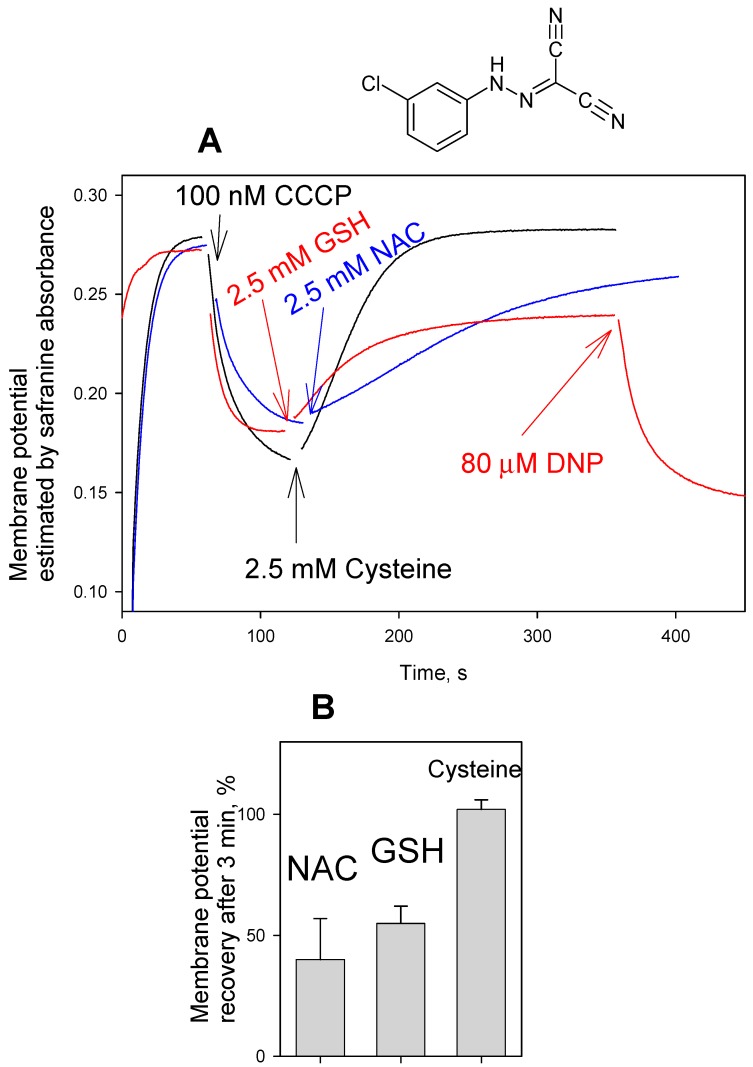
(**A**) Effect of N-acetylcysteine (NAC, 2.5 mM, blue curve), glutathione (GSH, 2.5 mM, red curve), or cysteine (2.5 mM, black curve) on the uncoupling activity of carbonyl cyanide 3-chlorophenylhydrazone (CCCP) (100 nM) in rat liver mitochondria estimated by the mitochondrial membrane potential measurements with safranine O (15 µM). Y-axis shows absorbance of safranine at 555 nm minus absorbance at 523 nm. (**B**) Mean ± S.D. (*n* = 3) of the recovery of mitochondrial membrane potential measured at 3 min. after the addition of 2.5 mM NAC, GSH, or cysteine (in % to the effect of CCCP). Chemical structure of CCCP is shown on the top of the Figure.

Figure 1 displays time courses of ΔΨ generation in isolated rat liver mitochondria (RLM), as monitored by absorbance changes of safranine O. It is seen that the safranine O response recorded upon the energization of RLM by succinate in the presence of rotenone decreased in a minute timescale after the addition of FCCP at a low concentration (20 nM), resulting in a partial loss of ΔΨ. However, the reversal of the FCCP effect was observed, if RLM were supplemented with NAC either before or after the FCCP addition (Figure 1A). Qualitatively similar changes in the ΔΨ kinetics were observed in the presence of FCCP, when glutathione (Figure 1B), cysteine (Figure 1C), or DTT (Figure 1D) were used instead of NAC. Figure 2B presents concentration dependences of NAC, GSH, and cysteine effect on the FCCP-induced decrease in mitochondrial membrane potential, while Figure 2A illustrates the typical ΔΨ recordings at various concentrations of cysteine. It is seen that cysteine was much more effective in reversing the depolarizing action of FCCP than GSH and NAC. Figure 3 shows that the thiol-containing compounds also caused the recovery of mitochondrial ΔΨ after CCCP-induced depolarization (Figure 3), with cysteine being considerably more potent than GSH and NAC. 

**Figure 4:**
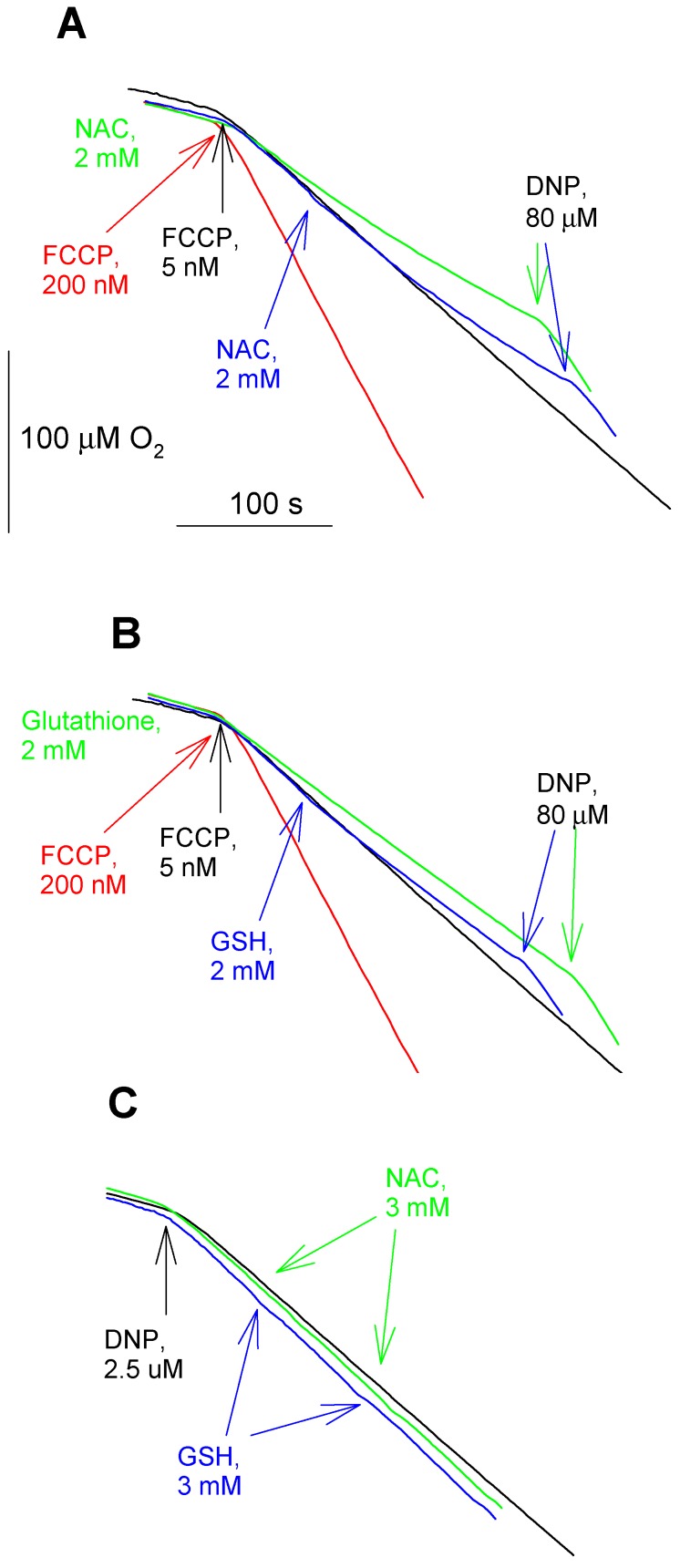
(**A**) Effect of N-acetylcysteine (NAC, 2 mM) on the stimulation of respiration of rat liver mitochondria by FCCP (5 nM). (**B**) Effect of glutathione (GSH, 2 mM) on the stimulation of respiration of mitochondria by FCCP (5 nM). Green curves corresponded to the addition of NAC (2 mM, **A**) or GSH (2 mM, **B**) prior to mitochondria. (**C**) The absence of the effect of NAC or GSH in the case of DNP-stimulated respiration. For other conditions, see Materials and methods.

Figure 4 shows the parallel measurements of the effect of the thiol-containing compounds on the FCCP-induced changes in mitochondrial respiration kinetics. The addition of NAC (Figure 4A), GSH (Figure 4B), cysteine or DTT (data not shown) reversed the accelerating effect of FCCP on the RLM respiration. The experimental curves in Figure 4 show representative data. Statistical analysis confirmed 20% ± 3% (*n* = 3) reduction of the respiration rate by 2 mM NAC, and 16% ± 2% (*n* = 3) reduction of the respiration rate by 2 mM GSH. 

**Figure 5:**
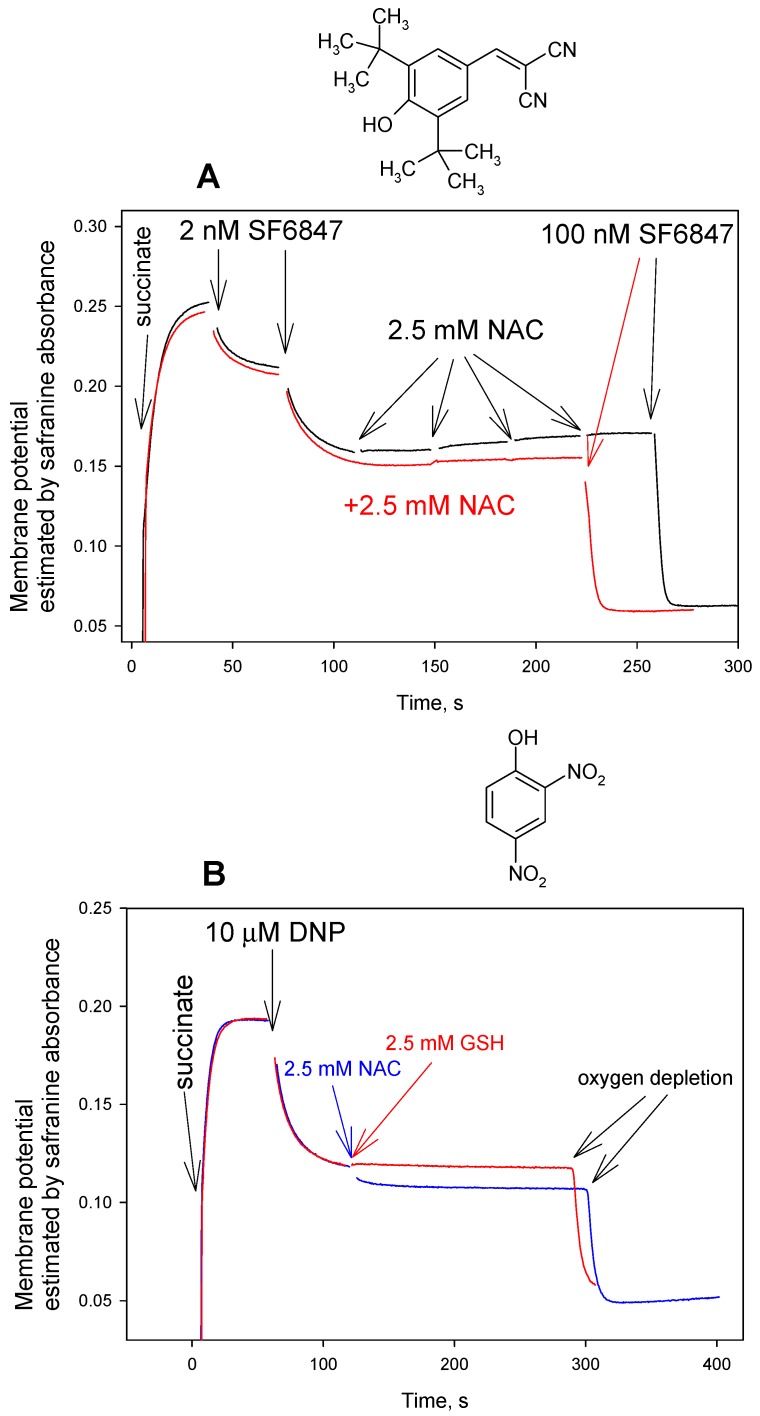
(**A**) Effect of N-acetylcysteine (NAC, 2.5 mM) on the uncoupling activity of SF6847 (4 nM totally) in rat liver mitochondria estimated by the mitochondrial membrane potential measurements with safranine O (15 µM). Y-axis shows absorbance of safranine at 555 nm minus absorbance at 523 nm. Red curve corresponded to the addition of NAC (2.5 mM) prior to mitochondria. For other conditions, see Materials and methods. Chemical structure of SF6847 is shown on top of the Figure. (**B**) Effect of N-acetylcysteine (NAC, 2.5 mM, blue curve) or glutathione (GSH, 2.5 mM, red curve) on the uncoupling activity of DNP (10 μM) in rat liver mitochondria. Chemical structure of DNP is shown on top of the plot. For other conditions, see Materials and methods.

According to the analysis of the ^13^C NMR spectrum of the reaction product of carbonyl cyanide phenylhydrazone with cysteine, the reaction represents addition to a nitrile group [32]. Based on this conclusion, it could be expected that the activity of tyrphostin A9 (3,5-Di-tert-butyl-4-hydroxybenzylidenemalononitrile), which is a very potent uncoupler that is usually called SF6847 in bioenergetics literature, is also sensitive to thiols. On the contrary, the results that are presented in Figure 5A reveal that NAC and other thiol compounds studied here (cysteine and GSH, data not shown) exerted a negligible, if any, effect on the SF6847-induced uncoupling of RLM. Importantly, all of these thiols were also ineffective in reversing the uncoupling action of DNP (Figure 4C and Figure 5B), as well as that of TTFB, niclosamide, and tetrachlorosalicylanilide (data not shown), both in the membrane potential and respiration rate measurements.

**Figure 6:**
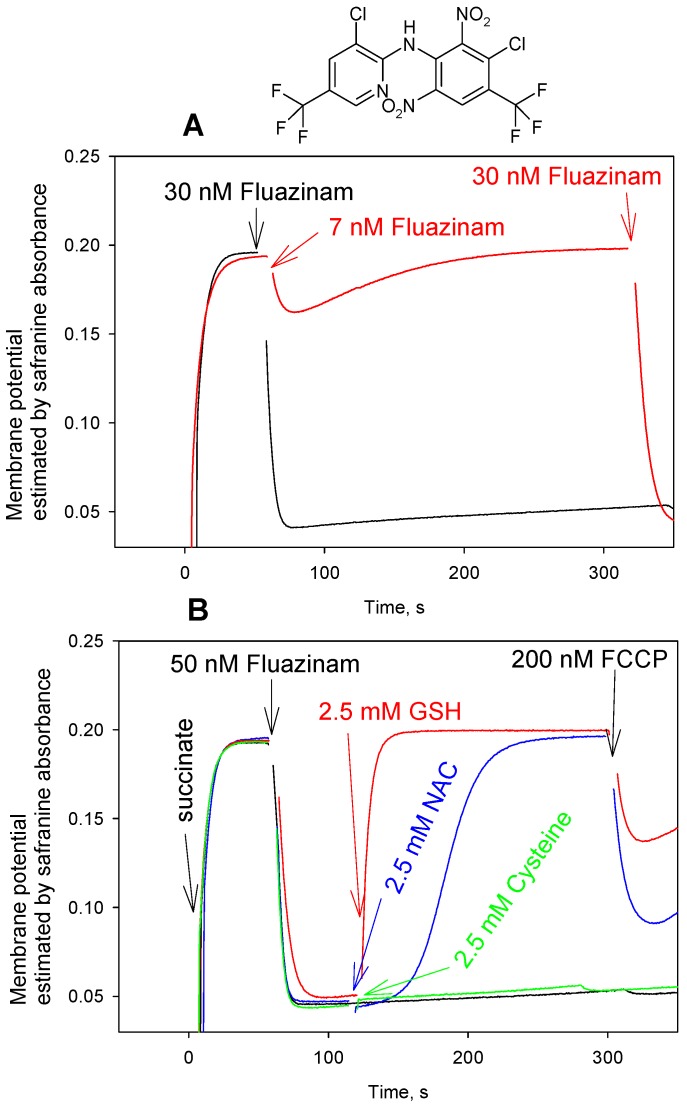
(**A**) Time courses of the mitochondrial membrane potential monitored by safranine O (15 µM) absorbance changes upon the addition of fluazinam at various concentrations. Y-axis shows absorbance of safranine at 555 nm minus absorbance at 523 nm. (**B**) Effect of N-acetylcysteine (NAC, 2.5 mM, blue curve), glutathione (GSH, 2.5 mM, red curve), or cysteine (2.5 mM, green curve) on the uncoupling activity of fluazinam at a high concentration (50 nM) in rat liver mitochondria. The chemical structure of fluazinam is shown on top of the Figure. For other conditions, see Materials and methods.

**Figure 7:**
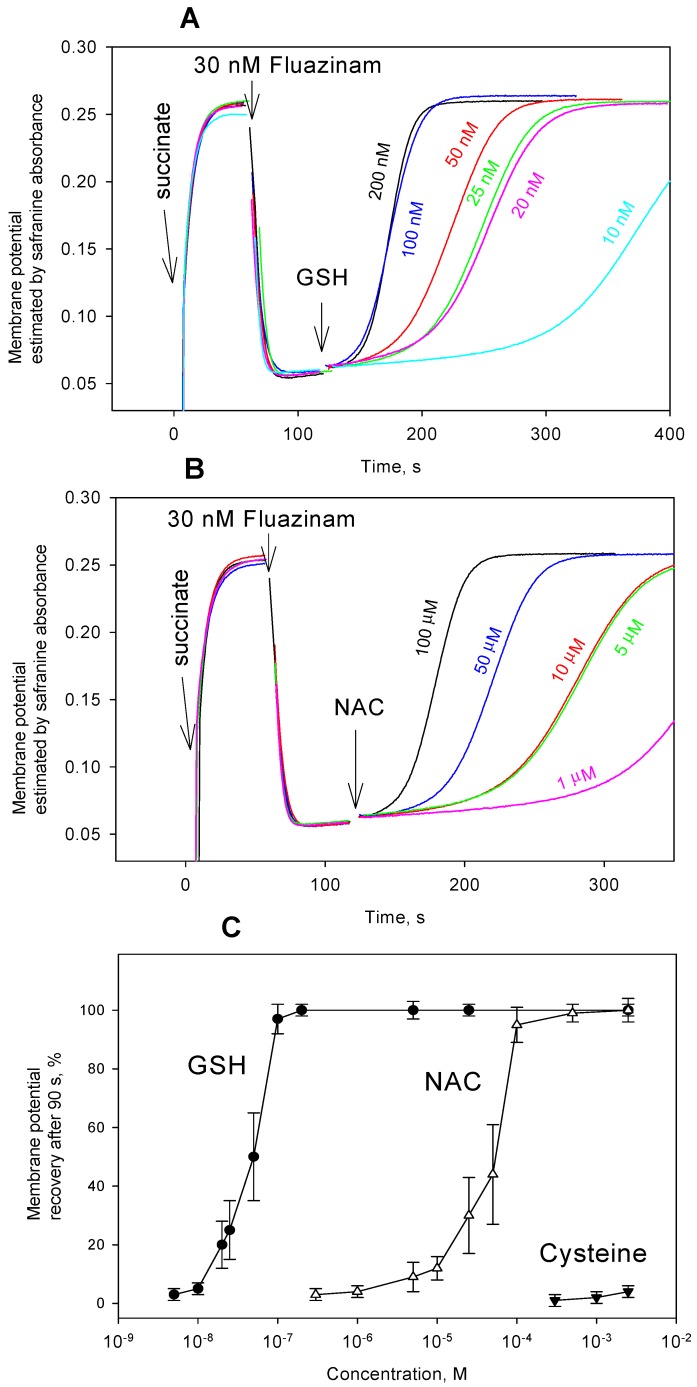
(**A**) Effect of various concentrations of GSH on the uncoupling activity of fluazinam (30 nM) in rat liver mitochondria estimated by the mitochondrial membrane potential measurements with safranine O (15 µM). Y-axis shows absorbance of safranine at 555 nm minus absorbance at 523 nm. (**B**) Effect of various concentrations of NAC on the uncoupling activity of fluazinam (30 nM) in rat liver mitochondria. (**C**) Dose dependence (shows Mean ± S.D., *n* = 3) of the recovery of the membrane potential 90 s after the addition of NAC, GSH, and cysteine. For other conditions, see Materials and methods.

Based on the earlier reported abrogation of the uncoupling activity of fluazinam in mitochondria that were ascribed to endogenous glutathione [25], it was of interest to probe the effect of the addition of thiol-containing compounds on the fluazinam-mediated uncoupling of RLM. In our hands, both accelerating respiration and decreasing membrane potential of RLM by fluazinam at nanomolar concentrations rapidly disappeared with time (Figure 6A). Increasing the concentration of fluazinam suppressed its deactivation, which is in line with [25]. As it is seen in Figure 6, fluazinam exhibited rather stable depolarizing activity at a concentration of 30 nM. The addition of either GSH or NAC under these conditions elicited the very fast recovery of mitochondrial membrane potential (Figure 6B), which was much faster than that seen with FCCP and CCCP. Surprisingly enough, no effect on the fluazinam-caused mitochondrial uncoupling was produced by cysteine (Figure 6B), which is the most active in the case of carbonyl cyanide phenylhydrazones. DTT was also practically ineffective with fluazinam (data not shown). Figure 7C demonstrates the concentration dependences of GSH, NAC, and cysteine effect on the fluazinam-induced decrease in ΔΨ, while Figure 7A,B illustrate the typical ΔΨ recordings at various concentrations of GSH and NAC. It is seen that GSH exerted the striking recoupling action, even at a concentration of 5 nM. The kinetics of the GSH effect accelerated in the concentration range from 5 nM to 100 nM, while that of NAC from 5 µM to 100 µM. The difference between the effects of the thiol compounds on fluazinam and those on FCCP and CCCP is apparently due to their structural diversity, thereby leading to different mechanisms of the interaction.

**Figure 8:**
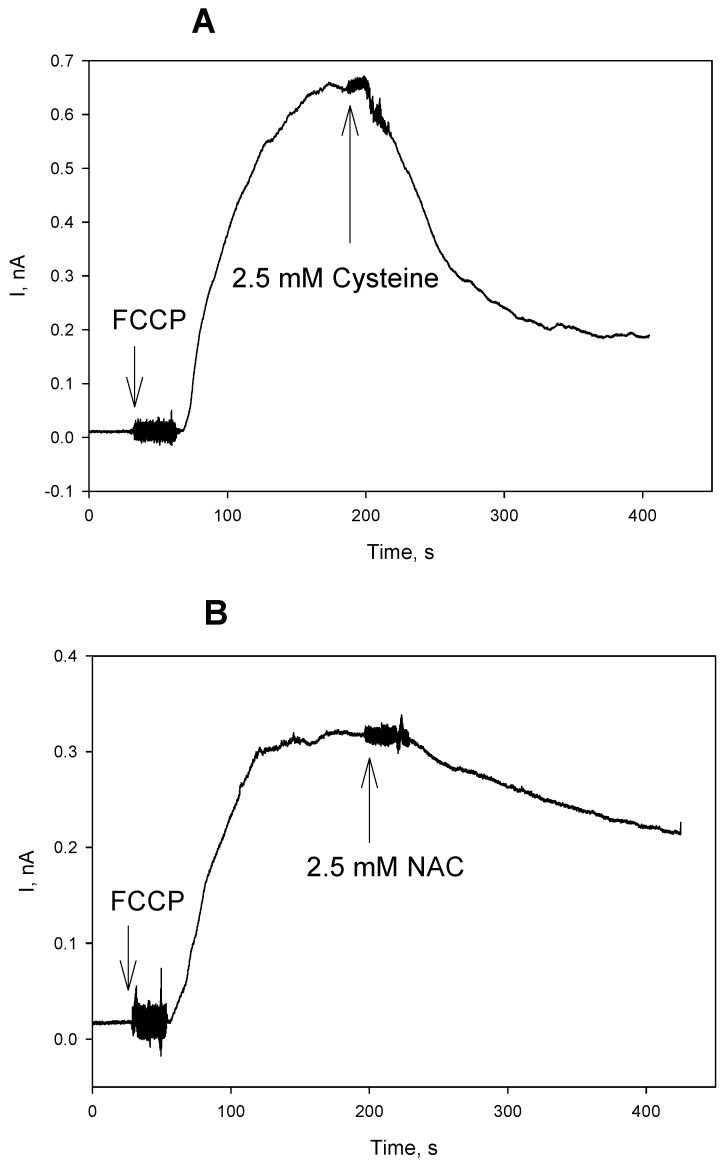
(**A**) Effect of cysteine (2.5 mM) on the FCCP (3 μM)-mediated electrical current through planar bilayer lipid membrane (BLM) made from DPhPC. (**B**) Effect of N-acetylcysteine (NAC, 2.5 mM) on the FCCP (3 μM)-mediated electrical current through the BLM. The solution was 50 mM Tris, 50 mM MES, 10 mM KCl, pH 7.4. The voltage applied to the BLM was 50 mV.

**Figure 9:**
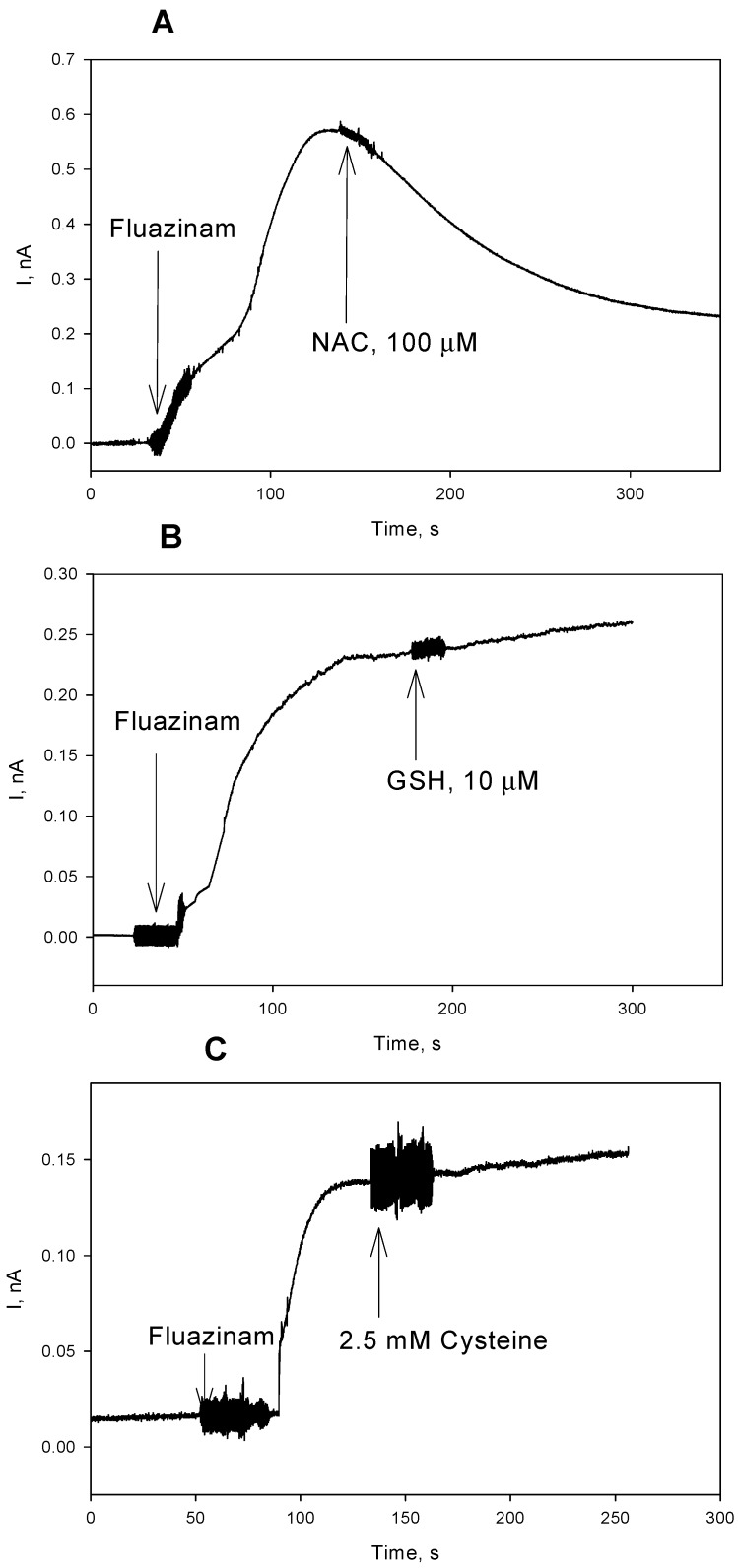
(**A**) Effect of N-acetylcysteine (NAC, 100 µM) on the fluazinam (3 μM)-mediated electrical current through planar bilayer lipid membrane (BLM) made from DPhPC. (**B**) Effect of GSH (10 µM) on the fluazinam (3 μM)-mediated electrical current through planar bilayer lipid membrane (BLM) made from DPhPC. (**C**) Effect of cysteine (2.5 mM) on the fluazinam (3 μM)-mediated electrical current through the BLM. The solution was 50 mM Tris, 50 mM MES, 10 mM KCl, pH 7.4. The voltage applied to BLM was 50 mV.

**Figure 10:**
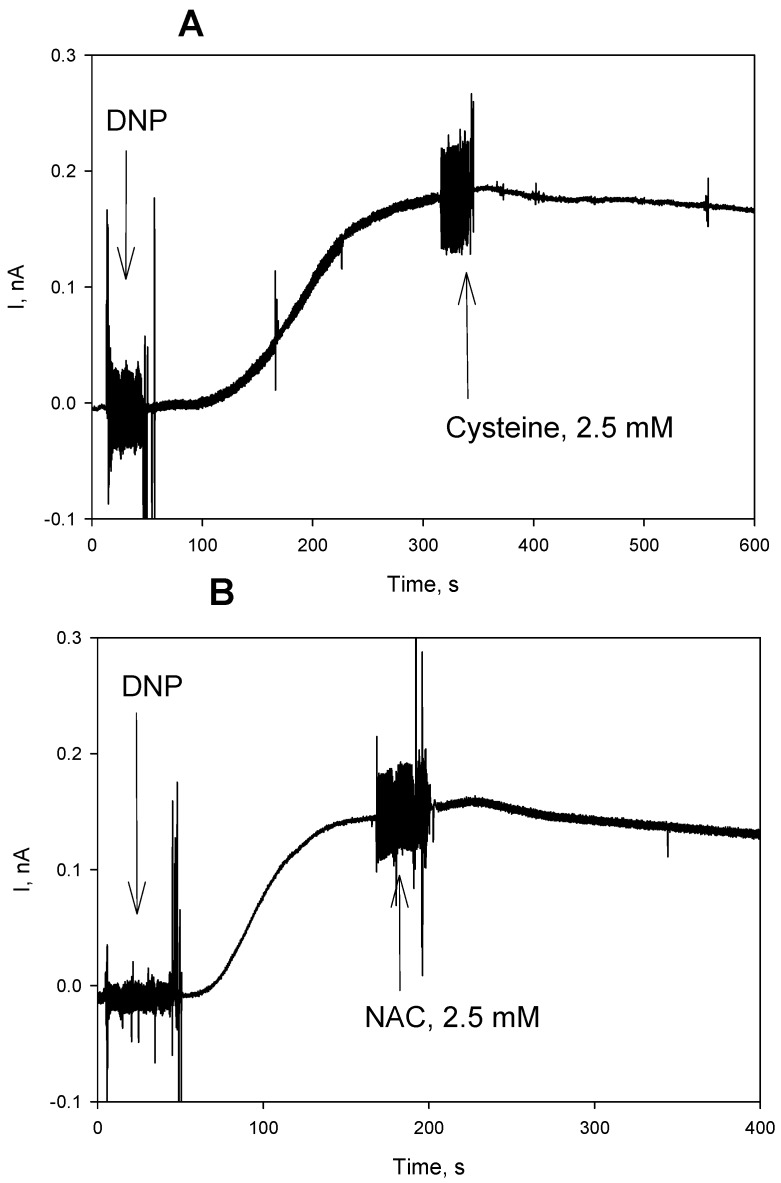
(**A**) Effect of cysteine (2.5 mM) on the DNP (500 μM)-mediated electrical current through planar bilayer lipid membrane (BLM) made from DPhPC. (**B**) Effect of N-acetylcysteine (NAC, 2.5 mM) on the DNP (500 μM)-mediated electrical current through the BLM. The solution was 50 mM MES, 10 mM KCl, pH 5.0. The voltage applied to BLM was 50 mV.

We tested an effect of the thiols on the protonophoric activity of the uncouplers in artificial BLM in further experiments. Figure 8 displays the time course of electrical current generated across BLM under voltage-clamp conditions after the addition of FCCP, reflecting its protonophoric activity. It is seen that the subsequent addition of cysteine at both sides of BLM caused a significant decrease in the current that is induced by FCCP (Figure 8A). Similar changes in the BLM current were observed with CCCP (data not shown). NAC caused a reduction of the FCCP-induced current with a slower kinetics (Figure 8B), which is in line with our observations on mitochondria (Figure 1). The experimental curves in Figure 8 show representative data. Statistical analysis confirmed 65% ± 14% (*n* = 3) reduction of the BLM current by 2.5 mM cysteine, and 25% ± 6% (*n* = 3) reduction of the BLM current by 2.5 mM NAC (2 min. after the addition of cysteine or NAC). A similar effect of NAC was also observed with fluazinam-induced BLM current (Figure 9A, 55% ± 9%, *n* = 3). On the contrary, GSH, being extremely potent in removing the fluazinam-mediated uncoupling of mitochondria (Figure 6 and Figure 7), did not inhibit the fluazinam-induced BLM current at the concentration effective in mitochondria (Figure 9B). Cysteine appeared to be completely ineffective with fluazinam on BLM (Figure 9C), as was found with mitochondria (Figure 6B). No effects of cysteine and NAC on the BLM current were observed in the experiments with DNP (Figure 10). Thus, the BLM study demonstrated the direct interaction of FCCP and CCCP with the thiol-containing compounds and the absence of this interaction for DNP.

The extraordinary sensitivity of the fluazinam action on the mitochondrial uncoupling to GSH in combination with the absence of the influence of GSH on the fluazinam-induced BLM current could be explained, when suggesting the involvement of a specific mitochondrial enzyme in the interaction of GSH with fluazinam in mitochondria. Mitochondrial glutathione S-transferase (GST) is likely to be the most probable candidate to catalyze the nucleophilic substitution of the 3-chlorine atom in fluazinam by a GSH residue based on an enormous body of evidence showing that halogenated nitrobenzene derivatives can serve as substrates of GST to generate GSH adducts via the displacement of a halogen atom with GSH [33,34]. According to [33], many diverse reactions that were catalyzed by GSTs may be formulated as a nucleophilic attack of enzyme-bound GSH on the electrophilic center of the second substrate. Actually, fluazinam has been found to be the most reactive towards GST of the agrochemicals tested in [35], even more effective than 1-chloro-2,4-dinitrobenzene, being commonly used as the standard second substrate of GST. Importantly, NAC could serve as an efficient substrate for GST instead of GSH [36], whereas neither cysteine nor dithiothreitol could substitute for GSH [37]. It can be speculated that fluazinam may react with NAC both with and without catalysis by GST while taking into account a decrease in the fluazinam-induced BLM current after the addition of NAC.

In contrast to the case of fluazinam, where the enzyme GST is suggested to be involved in the interaction of the uncoupler with GSH and probably with NAC, the carbonylcyanide phenylhydrazone derivatives FCCP and CCCP most likely undergo direct modification by the thiol-containing compounds without the participation of mitochondrial proteins. This assumption is supported by the suppression of the FCCP-induced BLM current upon the addition of cysteine (Figure 8A) and NAC (Figure 8B). 

Based on the results that were obtained, it can be concluded that examining the impacts of thiol-containing antioxidants on mitochondria-linked processes by using such popular uncouplers as FCCP, CCCP, and fluazinam should be considered with serious caution. For example, in [38], it was argued that mild uncoupling could increase or decrease mitochondrial ROS, depending on the redox environment. Actually, this statement was based on the reduction of the FCCP effect on the ROS level that is caused by the addition of DTT (1–2 mM). In view of our data, the FCCP concentration itself would have drastically decreased upon the addition of DTT in such experiments. 

## Abbreviations

carbonyl cyanide 4-(trifluoromethoxy)phenylhydrazone (FCCP), carbonyl cyanide 3-chlorophenylhydrazone (CCCP), 3,5-Di-tert-butyl-4-hydroxybenzylidenemalononitrile (SF6847, tyrphostin A9), 2,4-dinitrophenol (DNP), tetrachlorotrifluoromethylbenzimidazole (TTFB), N-acetylcysteine (NAC), glutathione (GSH), dithiothreitol (DTT), diphytanoylphosphatidylcholine (DPhPC), glutathione S-transferase (GST), rat liver mitochondria (RLM), bilayer lipid membrane (BLM), reactive oxygen species (ROS), mitochondrial membrane potential (ΔΨ).

## References

[1] Boveris A., Chance B. (1973). The mitochondrial generation of hydrogen peroxide. General properties and effect of hyperbaric oxygen. Biochem. J..

[2] Boveris A. (1977). Mitochondrial production of superoxide radical and hydrogen peroxide. Adv. Exp. Med. Biol..

[3] Cino M., Del Maestro R.F. (1989). Generation of hydrogen peroxide by brain mitochondria: the effect of reoxygenation following postdecapitative ischemia. Arch. Biochem. Biophys..

[4] Korshunov S.S., Skulachev V.P., Starkov A.A. (1997). High protonic potential actuates a mechanism of production of reactive oxygen species in mitochondria. FEBS Lett..

[5] Liu S.S. (1997). Generating, partitioning, targeting and functioning of superoxide in mitochondria. Biosci. Rep..

[6] Tan S., Sagara Y., Liu Y., Maher P., Schubert D. (1998). The regulation of reactive oxygen species production during programmed cell death. J. Cell Biol..

[7] Sagara Y., Ishige K., Tsai C., Maher P. (2002). Tyrphostins protect neuronal cells from oxidative stress. J. Biol. Chem..

[8] Starkov A.A., Fiskum G. (2003). Regulation of brain mitochondrial H_2_O_2_ production by membrane potential and NAD(P)H redox state. J. Neurochem..

[9] Sullivan P.G., Springer J.E., Hall E.D., Scheff S.W. (2004). Mitochondrial uncoupling as a therapeutic target following neuronal injury. J. Bioenerg. Biomembr..

[10] Geisler J.G., Marosi K., Halpern J., Mattson M.P. (2017). DNP, Mitochondrial Uncoupling and Neuroprotection: A Little Dab’ll Do Ya. Alzheimers Dement..

[11] Childress E.S., Alexopoulos S.J., Hoehn K.L., Santos W.L. (2018). Small molecule mitochondrial uncouplers and their therapeutic potential. J. Med. Chem..

[12] Brennan J.P., Southworth R., Medina R.A., Davidson S.M., Duchen M.R., Shattock M.J. (2006). Mitochondrial uncoupling, with low concentration FCCP, induces ROS-dependent cardioprotection independent of KATP channel activation. Cardiovasc. Res..

[13] Xiao B., Goh J.-Y., Xiao L., Xian H., Lim K.-L., Liou Y.-C. (2017). Reactive oxygen species trigger Parkin/PINK1 pathway-dependent mitophagy by inducing mitochondrial recruitment of Parkin. J. Biol. Chem..

[14] Zhang X., Cheng X., Yu L., Yang J., Calvo R., Patnaik S., Hu X., Gao Q., Yang M., Lawas M. (2016). MCOLN1 is a ROS sensor in lysosomes that regulates autophagy. Nat. Commun..

[15] Jing K., Shin S., Jeong S., Kim S., Song K.-S., Park J.-H., Heo J.-Y., Seo K.-S., Park S.-K., Kweon G.-R. (2014). Docosahexaenoic acid induces the degradation of HPV E6/E7 oncoproteins by activating the ubiquitin–proteasome system. Cell Death Dis..

[16] Park W.H., Han Y.H. (2011). Intracellular glutathione levels are involved in carbonyl cyanide p-(trifluoromethoxy) phenylhydrazone-induced apoptosis in As4.1 juxtaglomerular cells. Int. J. Mol. Med..

[17] Lee J.E., Kang J.S., Shin I.C., Lee S.J., Hyun D.H., Lee K.S., Koh H.C. (2011). Fluazinam-induced apoptosis of SH-SY5Y cells is mediated by p53 and Bcl-2 family proteins. NeuroToxicology.

[18] Chaudhari A.A., Seol J.W., Kim S.J., Lee Y.J., Kang H.S., Kim I.S., Kim N.S., Park S.Y. (2007). Reactive oxygen species regulate Bax translocation and mitochondrial transmembrane potential, a possible mechanism for enhanced TRAIL-induced apoptosis by CCCP. Oncol. Rep..

[19] Drobnica L., Sturdik E. (1979). The reaction of carbonyl cyanide phenylhydrazones with thiols. Biochim. Biophys. Acta.

[20] Toninello A., Siliprandi N. (1982). Restoration of membrane potential in mitochondria deenergized with carbonyl cyanide p-trifluoromethoxyphenylhydrazone (FCCP). Biochim. Biophys. Acta.

[21] Mlejnek P., Dolezel P. (2015). Loss of mitochondrial transmembrane potential and glutathione depletion are not sufficient to account for induction of apoptosis by carbonyl cyanide 4-(trifluoromethoxy)phenylhydrazone in human leukemia K562 cells. Chem. Biol. Interact..

[22] Heytler P.G. (1963). Uncoupling of oxidative phosphorylation by carbonyl cyanide phenylhydrazones. I. Some characteristics of m-Cl-CCP action on mitochondria and chloroplasts. Biochemistry.

[23] Blum J.J., Sanadi D.R. (1964). Activation of myosin adenosine triphosphatase by carbonyl cyanide p-chlorophenylhydrazone. J. Biol. Chem..

[24] De Kiewiet D.Y., Hall D.O., Jenner E.L. (1965). Effect of carbonylcyanide m-chlorophenylhydrazone on the photochemical reactions of isolated chloroplasts. Biochim. Biophys. Acta.

[25] Guo Z., Miyoshi H., Komyoji T., Haga T., Fujita T. (1991). Uncoupling activity of a newly developed fungicide, fluazinam [3-chloro-N-(3-chloro-2,6-dinitro-4-trifluoromethylphenyl)-5-trifluoromethyl-2-pyridinamine]. Biochim. Biophys. Acta.

[26] Brandt U., Schubert J., Geck P., von Jagow G. (1992). Uncoupling activity and physicochemical properties of derivatives of fluazinam. Biochim. Biophys. Acta.

[27] Hollingworth R.M., Gadelhak G.G. (1998). Mechanisms of action and toxicity of new pesticides that disrupt oxidative phosphorylation. Rev. Toxicol..

[28] Johnson D., Lardy H. (1967). Isolation of liver or kidney mitochondria. Methods Enzymol..

[29] Antonenko Y.N., Khailova L.S., Rokitskaya T.I., Nosikova E.S., Nazarov P.A., Luzina O.A., Salakhutdinov N.F., Kotova E.A. (2019). Mechanism of action of an old antibiotic revisited: role of calcium ions in protonophoric activity of usnic acid. Biochim. Biophys. Acta Bioenerg..

[30] Akerman K.E., Wikstrom M.K. (1976). Safranine as a probe of the mitochondrial membrane potential. FEBS Lett..

[31] Mueller P., Rudin D.O., Tien H.T., Wescott W.C. (1963). Methods for the formation of single bimolecular lipid membranes in aqueous solution. J. Phys. Chem..

[32] Sulo P., Sturdik E., Liptaj T., Jakubik T., Antalik M. (1985). Structure characterization of reaction products from phenylhydrazono-propandinitriles and thiols. Collect. Czechoslovak Chem. Commun..

[33] Keen J.H., Habig W.H., Jakoby W.B. (1976). Mechanism for the several activities of the glutathione S-transferases. J. Biol. Chem..

[34] Inoue K., Ohe T., Mori K., Sagara T., Ishii Y., Chiba M. (2009). Aromatic substitution reaction of 2-chloropyridines catalyzed by microsomal glutathione S-transferase 1. Drug. Metab. Dispos..

[35] Clarke E.D., Greenhow D.T., Adams D. (1998). Metabolism-related assays and their application to agrochemical research: Reactivity of pesticides with glutathione and glutathione transferases. Pestic. Sci..

[36] Weinander R., Anderson C., Morgenstern R. (1994). Identification of N-acetylcysteine as a new substrate for rat liver microsomal glutathione transferase. A study of thiol ligands. J. Biol. Chem..

[37] Kraus P. (1980). Resolution, purification and some properties of three glutathione transferases from rat liver mitochondria. Hoppe Seylers Z. Physiol. Chem..

[38] Aon M.A., Cortassa S., O’Rourke B. (2010). Redox-optimized ROS balance: A unifying hypothesis. Biochim. Biophys. Acta.

